# Atypical traumatic anterior shoulder instability with excessive joint laxity: recurrent shoulder subluxation without a history of dislocation

**DOI:** 10.1186/s13018-018-0791-4

**Published:** 2018-04-11

**Authors:** Sung-Jae Kim, Chong-Hyuk Choi, Yun-Rak Choi, Wonyong Lee, Woo-Seok Jung, Yong-Min Chun

**Affiliations:** 1 Department of Orthopaedic Surgery, Arthroscopy and Joint Research Institute, Severance Hospital, Yonsei University College of Medicine, CPO Box 8044, 50-1 Yonsei-ro, Seodaemun-gu, Seoul, 120-752 South Korea; 2 Department of Orthopaedic Surgery, Severance Hospital, Yonsei University College of Medicine, Seoul, South Korea

**Keywords:** Joint laxity, Shoulder, Bankart repair, Arthroscopy

## Abstract

**Background:**

No previously published studies have examined recurrent traumatic incomplete events in patients with excessive joint laxity. The purpose of this study is to investigate outcomes after arthroscopic stabilization for recurrent traumatic shoulder subluxation in patients with excessive joint laxity but no history of dislocation.

**Methods:**

This study included 23 patients with glenoid bone defects less than 20% who underwent arthroscopic stabilization of recurrent shoulder subluxation and were available for at least 2 years follow-up. Outcomes were assessed with the subjective shoulder value (SSV), University of California Los Angeles (UCLA) shoulder score, Rowe score, and sports/recreation activity level.

**Results:**

Postoperatively, overall functional scores improved significantly (*p* <  0.001), compared to preoperative scores: SSV improved from 49.1 to 90.4; Rowe score improved from 36.7 to 90.2; and UCLA shoulder score improved from 26.3 to 32.5. Patient satisfaction rate was 87% (20/23 patients). Sports/recreation activity level (return to premorbid activity level; grade *I* = 100% to grade IV = less than 70%) was grade I in 7 patients, grade II in 11, grade III in 3, grade IV in 2. The incidence of any glenoid bone defect was 61% (14/23 patients), and the mean glenoid bone defect size was 8%; among these 14 patients, 8 (35%) exhibited 15–20% glenoid bone defects. Instability reoccurred in 2 patients (9%) who had 15–20% glenoid bone defect.

**Conclusion:**

Despite excessive joint laxity, overall functional outcomes after arthroscopic stabilization of recurrent shoulder subluxation were satisfactory. However, arthroscopic Bankart repair may not be reliable in patients with excessive joint laxity plus a glenoid bone defect size of more than approximately 15%.

## Background

Typically, recurrent traumatic anterior shoulder instability begins with an initial traumatic anterior dislocation episode and is followed by repeated episodes of anterior dislocation. Even if the recurrent episodes are not complete dislocations, patients exhibit a positive apprehension sign with 90° abduction and 90° external rotation at the shoulder. The recurrent episodes, in turn, cause repeated contact and erosion between the anterior glenoid rim and posterolateral humeral head. Consequently, the bone defect within the glenoid may enlarge, which may require a glenoid reconstituting operation, such as the Latarjet procedure [1–4].

In some patients, however, the initial trauma episode and later recurrent episodes do not require manual reduction, and the shoulder reduces itself. This is particularly likely in patients with excessive joint laxity. We postulate that in these patients, the initial and subsequent episodes are not true dislocations, but subluxations, although the patient might think that his or her shoulder popped out and in spontaneously. Nevertheless, spontaneous reduction can occur in recurrent shoulder dislocations after an initial dislocation episode; with a substantial glenoid bone defect or Hill-Sachs lesion, the shoulder may dislocate and reduce with ease. However, this is different from recurrent subluxation in which the shoulder reduces spontaneously because it does not completely dislocate.

Recently, Owens et al. reported an interesting study of patients with first-time, traumatic anterior shoulder subluxation, in which these incomplete events yielded a high rate of Bankart and Hill-Sachs lesions [5]. As these authors note, many incomplete events, such as transient luxation or benign subluxation, may be encountered, especially in young patients. Nevertheless, no previously published studies have examined recurrent traumatic incomplete events in patients with excessive joint laxity.

The purpose of this study was to investigate outcomes after arthroscopic stabilization for recurrent shoulder subluxation in patients with excessive joint laxity and no history of dislocation. We hypothesized that despite excessive laxity, arthroscopic stabilization would produce satisfactory functional outcomes, and the incidence and severity of the glenoid bone defects would be low because of absence of a previous dislocation.

## Methods

### Study population

We defined “recurrent traumatic shoulder subluxation” as anterior shoulder instability with recurrent subluxation, but without any history of dislocation requiring manual reduction. From March 2008 to March 2014, 29 patients with excessive joint laxity underwent arthroscopic stabilization in our institute for recurrent shoulder subluxation (as defined above). All operations were performed by a single surgeon. The indications for surgery were recurrent traumatic anterior shoulder subluxation refractory to conservative treatment for at least 3 months, positive apprehension sign at 90° abduction and 90° external rotation of the shoulder (ABER position), and a glenoid bone defect of less than 20% on the en-face view during three-dimensional computed tomography (3D CT). The study inclusion criteria were recurrent anterior shoulder subluxation initiated by a traumatic subluxation episode, without any history of dislocation, and follow-up data available for at least 2 years postoperatively. The exclusion criteria were voluntary instability, concomitant posterior or multi-directional instability, concomitant rotator cuff tear requiring repair, engaging Hill-Sachs lesion requiring concomitant remplissage, or previous surgery on the affected shoulder. Finally, 23 patients who met the inclusion/exclusion criteria were included in this study. Patient data, including medical records and radiological imaging results, were retrospectively reviewed.

### Functional and radiological assessments

Functional assessments of the affected shoulder included the subjective shoulder value (SSV, patient’s self-estimate of the value of the affected shoulder as a percentage of the value of a normal shoulder [6]), University of California Los Angeles (UCLA) shoulder score, Rowe score, and sports/recreation activity level. For the sports/recreation activity level, patients rated their postoperative activity level as a percentage of their premorbid level: grade I, no limitation (100% of premorbid level); grade II, mild limitation (90% or more of premorbid level); grade III, moderate limitation (70% or more of premorbid level); grade IV, severe limitation (less than 70% of premorbid level) or an inability to return to the previous sports/recreation activity [7–9]. Active range of motion (ROM) included forward flexion in the scapular plane, external rotation with the elbow at the side, and external/internal rotation at 90° shoulder abduction. The presence of excessive joint laxity was defined as a score of 4 or higher according to the Beighton and Horan criteria [10] (Table 1).

**Table 1:** The Beighton and Horan criteria for generalized joint laxity

Passive dorsiflexion of the little finger beyond 90° Passive apposition of the thumb to the flexor aspect of the forearm Hyperextension of the elbows beyond 10° Hyperextension of the knees beyond 10° Forward flexion of the trunk, with the knee straight, so that the palms of the hands rested easily upon the floor	
A patient receives 1 point for the ability to perform each of the listed action. Generalized joint laxity is defined as a score ≥ 4	

Recurrence of instability was defined as a positive apprehension sign at 90° abduction and 90° external rotation or a subluxation or dislocation episode. Preoperative radiological evaluation included true anteroposterior (AP) and axillary X-ray views, magnetic resonance imaging (MRI) or MR arthrography (MRA), and 3D CT (SOMATOM Sensation 64, Siemens, Erlangen, Germany). The glenoid bone defect percentage was calculated on the glenoid en-face view of the 3D CT images using the AP distance from bare spot method [11] (distance from anterior glenoid rim to the glenoid bare spot) in the PACS system (Centricity PACS; GS Medical System Information Technologies, Milwaukee, WI, USA).

### Operative procedures

Under general anesthesia, the patient underwent arthroscopic Bankart repair in the lateral decubitus position, after application of 10 lbs of longitudinal traction. Viewed from the standard posterior portal, a low anterior working portal was created over the lateral half of the subscapularis tendon, after a trial using a spinal needle to determine appropriate placement. An 8.5 mm cannula (CLEAR-TRAC complete cannula system; Smith & Nephew, Andover, MA, USA) was then introduced. A superior portal around the anterolateral corner of the acromion was likewise created after a spinal needle trial. As we prefer to look down at the glenoid from above, we usually switched the viewing portal to the superior portal. A 5.5 mm (CLEAR-TRAC complete cannula system; Smith & Nephew, Andover, MA) cannula was subsequently introduced into the posterior portal for shuttle-relay. From the top of the glenoid via the superior viewing portal, the overall glenoid and labrum were inspected. Once the Bankart lesion was identified, an electrocautery device and osteotome were used to release the anteroinferior labrum from glenoid by the extent necessary to identify the subscapularis muscle. The mobility of the detached capsulolabral complex was assessed to determine whether it could be pulled up onto the glenoid rim to form an adequate anterior labral bumper after the repair. After preparation of the glenoid rim and neck, the first suture anchor was inserted onto the glenoid rim at the 5:00 or 5:30 clock-face position in the right shoulder and at the 7:00 or 6:30 clock-face position in the left shoulder. With the use of a suture hook (ConMed Linvatec, Largo, FL) for shuttle-relay or a suture passer (Scorpion; Arthrex, Naples, FL), sutures could be passed through the capsule approximately 1 cm inferior to the anchor insertion, for shifting the capsule in a superior direction. The knots were secured on the capsular side of the reconstructed labrum, not on the glenoid side. The second and third suture anchors were inserted in the same manner at the 4:00 or 4:30 clock-face position and 3:00 or 3:30 clock-face position, respectively, onto the glenoid rim in the right shoulder, and 8:00 or 7:30 clock-face position and 9:00 or 8:30 clock-face position, respectively, on the left.

### Postoperative rehabilitation

The affected shoulder was kept in a sling for 4 to 5 weeks postoperatively. Pendulum and self-assisted circumduction exercises were started on the day after surgery. Self-assisted passive ROM exercises (table sliding/stretching exercise and forward flexion in the supine position) were begun after 4 to 5 weeks postoperatively, preferably performed after or during a hot-tub bath or shower. During the first 6 weeks, external rotation more than 30° was restricted. After 6 to 8 weeks postoperatively, self-assisted active ROM exercises were begun. Isotonic strengthening exercises were started 3 months after surgery. After 6 months postoperatively, patients were permitted to gradually return to their premorbid level of sports/recreation activities. Because of their excessive joint laxity, all patients were educated regarding how to prevent recurrence of instability during contact or collision sports/recreation activities.

### Statistical analyses

Statistical analyses were performed using the SPSS program (version 21.0; IBM, Armonk, NY, USA). The paired *t* test was used to compare preoperative and postoperative continuous or continuous ranked data, such as SSV, functional shoulder score (Rowe and UCLA), and active ROM. Statistical significance was set at *p* <  0.05. All data are mean ± standard deviation, except where otherwise indicated.

## Results

### Patient demographics, arthroscopic findings, and concomitant procedures

The current study included 19 males and 4 females, and all of whom had excessive joint laxity. Their mean age was 22.4 ± 3.5 years (range, 18 to 30 years). The dominant arm was involved in 21 patients (81%). The mean duration of symptoms before surgery was 24.6 ± 6.7 months (range, 12 to 36 months). The mean follow-up period after surgery was 37.8 ± 9.8 months (range, 24 to 59 months).

**Fig. 1:**
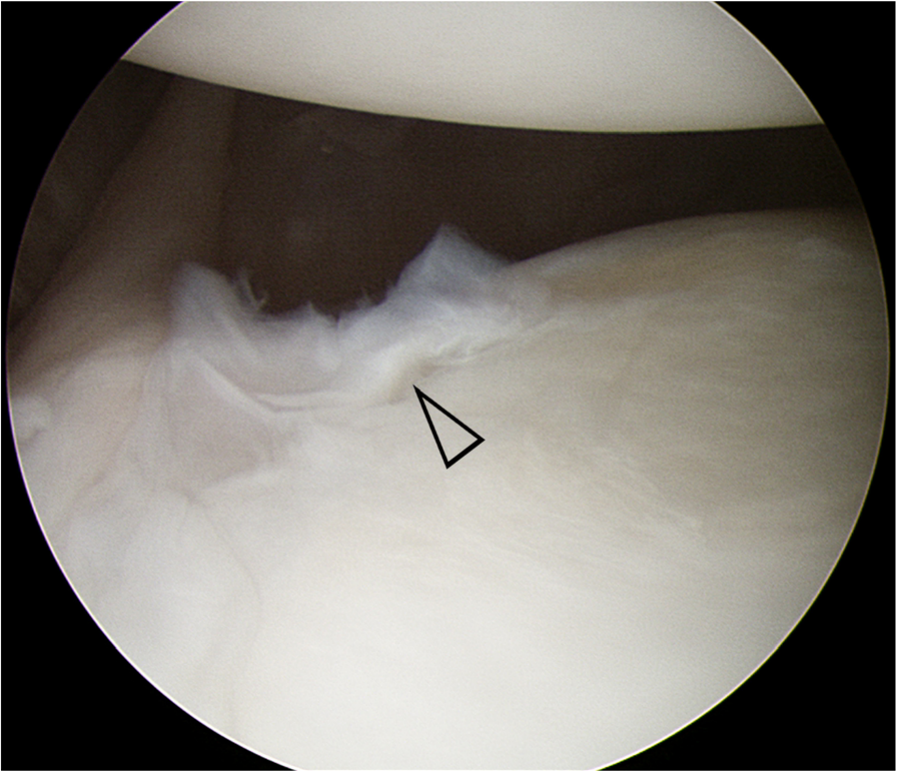
Incompletely torn and cracking anteroinferior labrum (open arrow head), viewed from the superior portal, right shoulder

A concomitant type II SLAP (superior labrum anterior to posterior) lesion was identified in 4 of the 23 patients (19%), which was repaired with suture anchors. A Bankart lesion was identified in 20 patients (87%). In the remaining three patients, anteroinferior labrum was incompletely torn and cracked (Fig. 1), but this differed from a typical Bankart lesion; in these patients, Bankart repair was not performed, but capsular shift and plication were performed with three non-absorbable sutures alone (without suture anchors). Unlike those patients with a Bankart lesion, these three patients also had no Hill-Sachs lesion. Four of the 23 patients (17%) exhibited mal-union or fibrous union of a bony Bankart lesion. The mean number of inserted suture anchors was 3.0 (range, 0 to 4).

### Clinical and radiological outcomes

At final follow-up, the overall functional scores improved significantly compared to preoperative values: SSV improved from 49.1 to 90.4 (*p* <  0.001); Rowe score improved from 36.7 to 92.2 (*p* <  0.001); and UCLA shoulder score improved from 26.3 to 32.5 (*p* <  0.001) (Table 2). The patient satisfaction rate was 87% (20 of 23 patients). The sports/recreation activity level (return to premorbid level of activities) was grade I in 7 patients, grade II in 11 patients, grade III in 3 patients, and grade IV in 2 patients. Recurrence of instability occurred in 2 of the 23 patients (9%) while participating in sports; one experienced a subluxation episode, and the other exhibited a positive apprehension sign but no subluxation or dislocation episode. There were no clinically significant differences between preoperative and postoperative values for active ROM, despite the presence of statistically significant difference: 174° vs. 171° in forward flexion (*p* = 0.005), 97° vs. 95° external rotation with the elbow at the side (*p* = 0.022), 120° vs. 116° external rotation at 90° abduction (*p* = 0.008), and 92° vs. 90° internal rotation at 90° abduction (*p* = 0.022) (Table 2).

**Table 2:** Shoulder functional scores (subjective shoulder value (SSV), ROWE score, and University of California Los Angeles (UCLA) shoulder scores) and active ranges of motion

	Preoperative	Final follow-up	*p* value
SSV	49.1 ± 10.4	90.4 ± 11.4	< 0.001
ROWE score	36.7 ± 6.8	92.2 ± 14.0	< 0.001
UCLA shoulder score	26.3 ± 1.3	32.5 ± 2.8	< 0.001
Forward flexion	174.3° ± 5.1°	171.3° ± 4.6°	0.005
External rotation with the elbow at the side	97.0° ± 8.2°	94.8° ± 9.5°	0.022
External rotation at 90° abduction	119.6° ± 7.1°	116.1° ± 8.9°	0.008
Internal rotation at 90° abduction	91.7° ± 4.9°	89.6° ± 5.6°	0.022

The values are given as the mean and standard deviation

The mean glenoid bone defect size was 8.0 ± 7.5%. Nine patients exhibited no glenoid defect, 6 patients had a 0–15% glenoid defect, and the remaining 8 patients exhibited a 15–20% glenoid defect. All 2 patients with instability recurrence had a 15–20% defect.

## Discussion

Despite their excessive joint laxity, patients in the current study achieved satisfactory overall functional outcomes after arthroscopic stabilization of their recurrent shoulder subluxation. Approximately, 80% were able to return to 90% or more of premorbid level of sports/recreation activities (grade I or II rating); 87% (20 of 23 patients) were satisfied with their outcomes, and the failure rate was 9% (2 of 23 patients).

Although the frequency of glenoid bone defects in recurrent shoulder instability in previous studies has ranged from 15 to 90% [12–15], the incidence of glenoid bone defect in our series was 61% (14 of 23 patients). Among these 14 patients, 8 exhibited a 15–20% glenoid bone defect. This was a higher frequency of substantial defects than we expected. We had surmised that patients with excessive joint laxity but no previous dislocation may have sufficient space and be able to avoid severe bone-on-bone erosion between the glenoid and humeral head, thereby avoiding substantial glenoid bone defects. However, our findings indicate that even recurrent subluxation can yield anteroinferior glenoid erosion in 61% of patients. Although the overall mean glenoid bone defect size was just 8%, and 39% of our patients had no glenoid defect, our observation that 35% of patients had 15–20% glenoid bone defect is important, particularly in the setting of excessive joint laxity, which may affect prognosis [8]. In our series, recurrent instability occurred in 2 patients (9%), all of whom had 15–20% defects. As these were 2 of the 8 patients with 15–20% glenoid bone defects, the incidence of recurrence in this glenoid defect range was 25%.

According to the literature, glenoid bone defects in the 15–25% range generally represent a borderline or approximate upper limit for using arthroscopic stabilization to treat recurrent shoulder instability [3, 16, 17]. However, a few studies have heretofore addressed arthroscopic stabilization for these borderline glenoid defects [8, 18]. Kim et al. reported their outcomes after arthroscopic stabilization for recurrent shoulder dislocation in patients with moderate (20–30%) defects [8]. Although that study was limited to patients with moderate to low functional demand or expectations for sports activity, the recurrence rate was 23% despite activity modification after surgery, suggesting that arthroscopic stabilization may not be reliable for patients with excessive joint laxity plus a glenoid bone defect of this size.

In 2007, Balg and Boileau described an “instability severity index score,” which is a 0 to 10 scale with higher numbers representing greater instability [19]. These authors concluded that arthroscopic Bankart repair was contraindicated if the score was over 6. The same group recently indicated an instability severity index score of 3 or less was most appropriate for arthroscopic Bankart repair [20]. Although our patients exhibiting 15–20% glenoid defects were considered to be appropriate candidate for arthroscopic stabilization, even using the stricter criteria, our findings that 25% developed recurrent instability suggested that glenoid arc reconstituting procedures might have produce better outcomes.

In the recent literature, the issues of shoulder subluxation and recurrent subluxation have not been well addressed. Owens et al. reported a study evaluating the pathoanatomy of traumatic shoulder subluxation, which was somewhat similar to our study [5]. Although our patients had recurrent subluxation, which is different from the Owens et al. patients, shoulder instability was triggered by an initial traumatic subluxation event in both studies. Considering the high rate of Bankart and Hill-Sachs lesions in both studies, subluxation might be a transient luxation in which extent of movement of the humeral head was sufficient to create pathologic changes but reduced spontaneously, rather than benign subluxation in which structural lesions were absent. However, even in transient luxation, Bankart lesions are not always created. Only incomplete tears and cracking in the labrum were noted in 13% of our patients. In Owens et al.’s study, 1 of 14 patients (7%) who underwent arthroscopic Bankart repair had cracking and scuffing of the labrum instead of a Bankart lesion [5]. Although the authors did not indicate whether that patient had excessive joint laxity, we surmise that in the setting of excessive joint laxity, transient luxation and subsequent recurrent subluxation may occur without a typical Bankart lesion. Recurrent shoulder subluxion has been discussed in some studies in the distant past [21–23]; however, in these studies, patients with and without Bankart lesions were mixed, and although some patients had excessive joint laxity, it is unclear whether the laxity definition was identical to that used in the current study.

In some previous reports, recurrent shoulder subluxation is regarded not as a post-traumatic instability, but rather as a multi-directional instability without trauma [5, 24, 25]. These reports noted that patients with excessive joint laxity tended to experience subluxation, rather than dislocation, in the absence of structural lesions. Patients with laxity might not have a pathologic lesion if subluxation is benign, as described above. However, if these patients experience transient luxation with or without trauma, they may develop the type of lesion noted in our series. Accordingly, it seems that recurrent shoulder subluxation can occur both with and without traumatic episodes.

Our study has limitations. First, although recurrent shoulder anterior subluxation with excessive joint laxity is relatively rare, this study included a limited number of patients and did not include a control group. Second, our study involved a relatively short period of follow-up after surgery. Considering that the mean age of our cohort was 22 years and the patients all had excessive joint laxity, it is possible that the rate of recurrence of instability may increase over time.

## Conclusion

Despite excessive joint laxity, overall functional outcomes after arthroscopic stabilization of recurrent shoulder subluxation were satisfactory. However, arthroscopic Bankart repair may not be reliable in patients with excessive joint laxity plus a glenoid bone defect size of more than approximately 15%.

## Abbreviations

3D CT: Three-dimensional computed tomography
ABER: 90° abduction and 90° external rotation of the shoulder
ROM: Range of motion
SLAP: Superior labrum anterior to posterior
SSV: Subjective shoulder value
UCLA: University of California Los Angeles
